# Worms love Coffee too! Characterizing the neural substrates that regulate odor-guided responses to coffee

**DOI:** 10.17912/micropub.biology.001242

**Published:** 2024-10-18

**Authors:** Ashley Vega, Alexis Chua, Annabelle Tran, Amber Seader, Emily Chang, Liz Ayala, Adriana Torres, Gianina Pontrelli, Gareth Harris

**Affiliations:** 1 California State University, Channel Islands, Camarillo, California, United States; 2 Jules Stein Eye Institute, University of California, Los Angeles, Los Angeles, California, United States

## Abstract

The coffee industry reaches over 80 billion US dollars in revenue partially due to the numerous chemicals that allow for coffee's highly attractive aroma and overall flavor. Many people integrate coffee into their everyday routine; therefore, understanding the attraction to coffee can facilitate, 1) the characterization of its attractive nature, and, 2) allow further understanding of how humans interpret taste and smell on a molecular and cellular level, from initial sensation to higher processing of these complex neural signals. We report that the model worm, *C. elegans* , can smell and perform strong attraction behavior using chemotaxis towards various types of coffee odors. In this study, we show that the nematode *C. elegans* is strongly attracted to various forms of coffee. We have also identified neuronal molecules that mediate this sensory-dependent behavior. Overall, we provide a platform to more thoroughly dissect the mechanisms and neuronal circuits that mediate odor-guided behavior to a complex human-sensed stimulus.

**Figure 1. Wild type worms are strongly attracted to coffee odor in different forms and use specific sensory transduction mechanisms:**
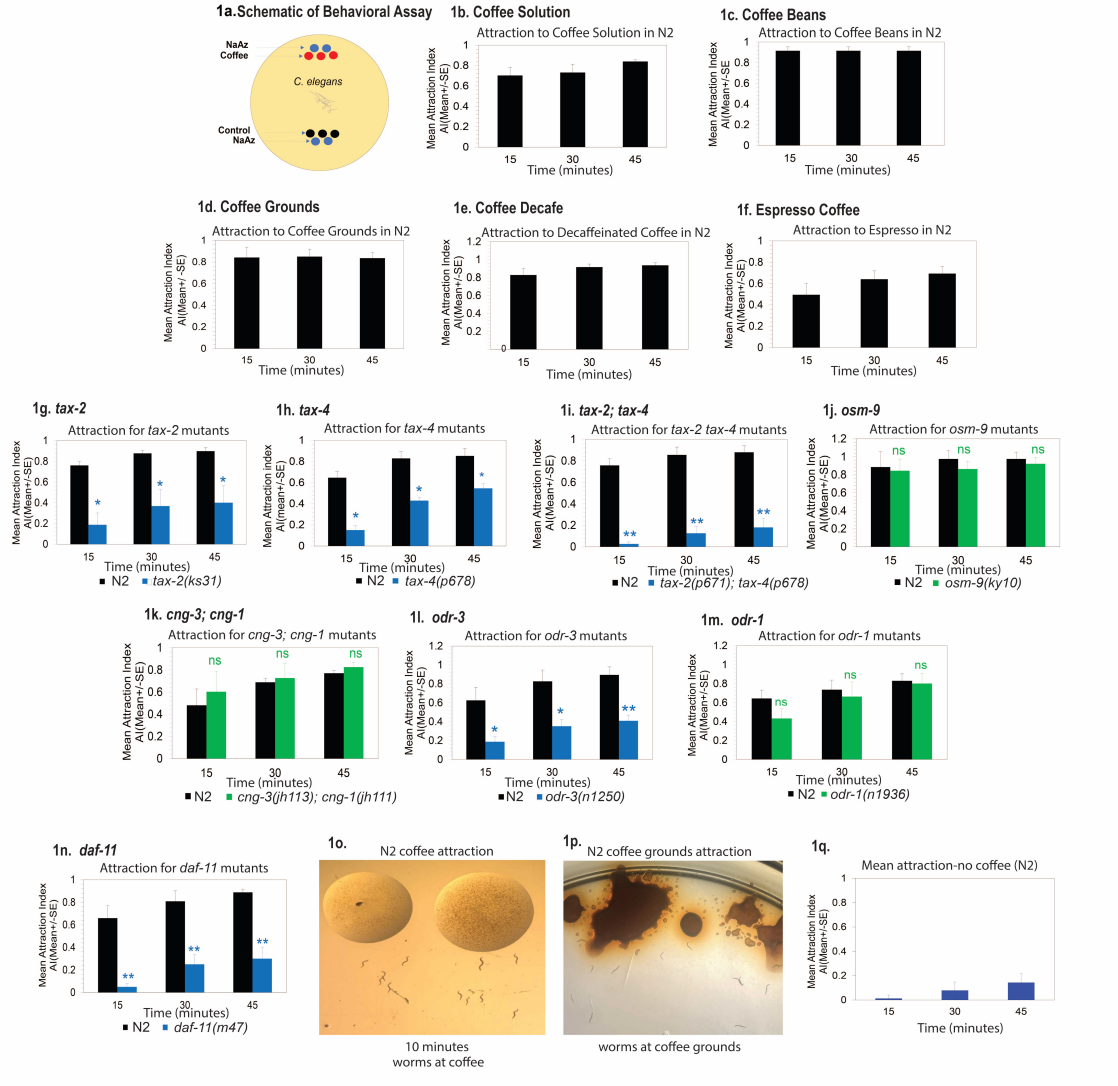
Figure 1 a) Schematic diagram of behavioral assay: chemotaxis assay for worms. Worms placed in the center of a 10 cm (100 mm) NGM agar plate. Number of worms recorded at attractant (coffee) and control at 15, 30, and 45 minute time points after application of coffee to the chemotaxis plate (Attraction index calculated as described in methodology). Wild type worms were examined for attraction to coffee solution (dark roast) (b) n=7 trials, coffee beans (c) n=4 trials, coffee grounds (d) n=4 trials, decaffeinated coffee (e) n=5 trials, espresso (f) n=4 trials. Attraction to coffee solution was compared between wild type and genetic mutants for *tax-2(ks31)* (g) n=4 trials, *tax-4(p678)* (h) n=4 trials, *tax-2(p671);tax-4(p678)* (i) n=5 trials, *osm-9(ky10)* (j) n=3 trials, *cng-3(jh113);cng-1(jh111)* (k) n=4 trials, *odr-3(n2150)* (l) n=3 trials, *odr-1(n1936)* (m) n=4 trials, *daf-11(m47)* (n) n=5 trials. o) Image of wild type worm population at coffee, 10 minutes after coffee exposure (dark roast coffee solution exposure in wild type N2 worms shown). p) Image of wild type worm population at coffee grounds after 10 minutes exposure (wild type N2 worms shown). q) Control experiment showing chemotactic index without any coffee present on the plate (containing water only) across 45 minutes (15, 30 and 45) showed no obvious bias in N2 worms, n=7 trials (blue bars). Note: For all graphs examining mutant animals, black bars= N2, green bars=mutants showing normal behavior, blue bars = defective mutants when compared to wild type N2. For all data analysis, Mean±SEM. A Mann-Whitney U Test was performed across each time point for all N2 vs mutant comparison tested on same day in parallel conditions, p ≤ 0.05*, p ≤ 0.01**, p ≤ 0.001***. ns=not statistically significant when comparing N2 and mutant genotype. For all assays in each panel, trials were performed on different days and consisted of 50-100 animals.

## Description

Coffee consumption in humans is due in part to its attractive aroma and flavor. Coffee odors and taste perception are often coupled with caffeine, and it has been shown that regular coffee consumers are more sensitive to caffeine-associated smells than their non-consumer counterparts (Parker et al., 2019). However, caffeine does not significantly affect coffee odor identification and threshold (Orgill et al., 2020; Meusel et al., 2016; Han et al., 2020). The number of chemicals sensed by an individual is correlated to threshold, discrimination, and identification status, as well as odor intensity demonstrating variability in coffee odor perception (Crnjar et al., 2023) . A number of studies show coffee is attractive to humans and select invertebrates, including Mediterranean fruit flies (Prokopy et al., 1996; Prokopy et al., 1997) . Despite this work, the underlying cellular mechanisms that mediate responses to coffee odor are not fully understood.

Here we investigate whether the nematode *C. elegans* is attracted to coffee. Previous studies have investigated *C. elegans* behavior in the presence of coffee, tea and other human consumed products, but none have addressed chemotactic responses (Urushihata et al., 2016; Min et al., 2017; Du et al., 2018; Zhang et al., 2022). In addition, worms have been treated with caffeine to assess behavior, for example, assessing the effects on worm longevity, behavioral plasticity, locomotion, and growth in the presence of caffeine (Kuo-Esser et al., 2023; Min et al., 2021; Manalo and Medina, 2020) . We use a plate chemotaxis assay that measures populations of adult worms in response to presented coffee odors (Ramos et al., 2020; Ramos et al., 2022) . We report that wild type worms are attracted to coffee.

To examine chemosensory behavior to coffee, we used a standard chemotaxis assay (Sengupta et al., 1996; Troemel et al., 1997; Bargmann et al., 1993; Bargmann et al., 1991; Wes and Bargmann, 2001) . We placed young adult wild type C. *elegans* on a chemotaxis plate in the presence of coffee solution ( Figure 1 a). We examined chemotaxis behavior based on attraction or repulsion to coffee drops placed at one end of the chemotaxis plate ( Figure 1 a-d). Wild type animals show high attraction to coffee ( Figure 1 a/b, image 1o). We also examined attraction to coffee in different forms, such as coffee grounds, espresso and coffee beans. Worms showed strong attraction to all of these ( Figure 1 c-f, image 1p). To address if the attraction to coffee was due to the presence of caffeine, we examined attraction to decaffeinated coffee ( Figure 1 e). Worms showed attractive behavior to decaffeinated coffee with similar index as coffee ( Figure 1 e). This suggests that coffee attraction in *C. elegans* is due to chemicals other than caffeine. We finally confirmed as a control that performing a chemotaxis assay with no coffee at either side of the plate (using water only) showed no behavioral bias ( Figure 1 q).

To dissect the neural pathways that control coffee odor attraction, we examined genetic mutants that lack known sensory transduction genes involved in sensory behavior (Bargmann et al., 1991; Ward et al., 2008; Ferkey et al., 2020). We examined mutants that lack ion channels, including cGMP-gated cation channels ( TAX-2 /4), transient receptor potential villanoid channels ( OSM-9 ), and other cyclic nucleotide-gated channels ( CNG-1 /3) (Tobin et al., 2002; Komatsu, et al., 1996; Coburn and Bargmann, 1996; Ferkey et al., 2020; Gray et al., 2004; Harris et al., 2014; Cho et al., 2004; He et al., 2016; Figure 1 ). These genes were chosen based on their known expression in select sensory neurons and roles in sensing different types of odorants (de Bono and Maricq, 2005, review). *osm-9 ( ky10* ) null mutants showed wild type attraction to coffee ( Figure 1 j). *tax-2 ( ks31 )* and *tax-4 ( p678 )* reduction of function mutants show a reduced attraction ( Figure 1 g-h, Coburn and Bargmann, 1996; Komatsu et al., 1996). In addition, *tax-2 ( p671 ); tax-4 ( p678 )* double mutants show a steep reduction in attraction ( Figure 1 i). *cng-3 ( jh113 ); cng-1 ( jh111 )* mutants show wild type attraction to coffee, suggesting no role in coffee attraction ( Figure 1 k, Shinkai et al., 2011; He et al *.* , 2016). Taken together, this reveals a role for cGMP-gated ion channels in coffee sensation.

We next examined mutants lacking G-protein alpha subunits (Jaansen et al *.,* 1999; Laans et al., 2004; Zwaal et al., 1997). Mutants lacking *odr-3* , a Gi/o alpha subunit (Royaie et al., 1998), showed reduced attraction to coffee, suggesting that G-protein signaling is important for coffee chemoattraction ( Figure 1 l). *odr-3* has been previously implicated in multiple sensory behaviors (Royaie et al., 1998; Harris et al., 2014; Ramos et al., 2022).

We then examined additional G-protein-dependent signaling genes. Two receptor guanylate cyclase (rGCs) genes, *odr-1* and *daf-11* , that are known to be expressed in sensory neurons required for sensation of different stimuli (L' etoille et al., 2001; Birnby et al., 2000; Lui et al., 2010; Harris et al., 2014). *odr-1 ( n1936 )* mutants were normal for coffee attraction ( Figure 1 m). *daf-11 ( m47 )* mutants showed reduced attraction to coffee ( Figure 1 n). Overall, we have characterized a new odor that is attractive to *C. elegans* and have identified specific genes encoding cGMP-gated channels, G-protein-alpha subunits and receptor guanylate cyclases (rGCs) that are involved in coffee attraction.

## Methods

Wild type worms were reared as previously described (Brenner et al., 1974; Hart et al., Wormbook, 2000) . Wild type worms were cultivated on *E. coli* OP50 standard lab food source prior to testing in the chemotaxis assay. Wild type ( N2 ) worms (CGC, Minnesota) were used in all chemotaxis assays and compared to all mutants tested in parallel. 10 cm (100 mm) plates (Thermofisher) were used for behavioral assays with no *E. coli* OP50 food present on the chemotaxis assay plate.

**Worm attraction assays**

Chemotaxis assay plates are NGM (nematode growth media) prepared two days before each assay. Approximately 50 – 100 wild type young adult worms were tested in each population assay. Briefly, NGM chemotaxis plates used for the assay were left at room temperature on the day of the behavioral assay. Wild type worms are washed three times in S. basal solution (As described in Bargmann et al., 1993; Bargmann and Horvitz, 1991; Troemel et al., 1997). The remaining worm pellet was added through pipetting to the center of the chemotaxis assay plate as seen in figure 1a. For select assays, wild type worms were initially transferred from an *E. coli* food plate to a transfer plate(non food plate-to allow food to be removed from worms) and then transferred to a drop of S. basal in the center of the chemotaxis plate. Once the worm pellet had dried on the chemotaxis assay plate (5 minutes), 3 drops of 2 microliter coffee was added to one side of the chemotaxis plate (as shown in figure 1a, schematic assay diagram). A control sample of water was added to the opposite side of the assay plate. Small drops of sodium azide (1 microliter at 1M) were used to paralyze worms either at the coffee source or control source. Sodium azide was placed immediately next to odor spots. Worms in the center of the plate at the beginning of the assay will be left to analyze their chemotaxis over multiple time points (15, 30, and 45 minutes after worms were added to assay plate), which will either be analyzed for attraction, repulsion or no bias to the coffee solution. To determine any attraction or avoidance, index's were generated based on calculating distribution of worms across NGM agar assay plates at 15, 30, and 45 minutes after coffee addition (see Figure 1 a-l). To determine attraction index throughout the assay with wild type and mutant animals (Bargmann et al., 1993) , total number (#) of worms paralyzed at odor source (paralyzed at coffee) - total number (#) of worms paralyzed at control sample on opposite side of the assay plate/total number (#) of worms on the whole chemotaxis assay plate counted at the beginning of the assay (i.e. includes all worms at attractant (coffee), control (no coffee) and worms that did not make either point source (other)). For the threshold for a worm to be counted at either side of the plate, worms were only counted as being at either side of the plate if the worms were paralyzed. Attraction index values range from 0 to +1.0, 0 being no attraction, and increasing positive number representing stronger attraction (Bargmann et al., 1993; Troemel et al., 1997 and Wormbook.org ), for 1b-1o graphs. For coffee beans: two beans were added per experiment to one end of the chemotaxis plate. For coffee grounds: a small amount of coffee grounds were placed at one end of the chemotaxis plate. Sodium azide was placed next to coffee beans or coffee grounds as normal to paralyze worms at the attractant. For solvent control assays on NGM agar plates, multiple drops of water were placed at each end of the assay plate and index's were calculated over 15, 30 and 45 minutes to confirm no bias observed without coffee (7 days repeated for wild type N2 worms in this control).

All animals were compared to wild type animals tested in parallel on the same day. Mean+/-SE, was determined per experiment and a Mann-Whitney U Test was performed to compare wild type N2 vs mutant, p ≤ 0.05*, p ≤ 0.01**, p ≤ 0.001***, to allow examination of significant differences between wild type and mutant worm strains at each time point (15, 30, and 45 minutes) tested on same day. For all assays in each panel, n=number of trials that were performed on different days and consisted of 50-100 animals.

## Reagents

**Strain list for present study:**

Wild type N2 (Bristol), FK104 *tax-2 ( ks31 )* , PR678 *tax-4 ( p678 )* , CX10 *osm-9 ( ky10 )* , BR5514 *tax-2 ( p671 ); tax-4 ( p678 )* , DR47 *daf-11 ( m47 )* , CX2065 *odr-1 ( n1936 )* , CX2205 *odr-3 ( n2150 )* , KJ5560 *cng-3 ( jh113 ); cng-1 ( jh111 ).*

**Reagents:**

Coffee samples/types used in present study: Dark roast coffee solution (Caffe Verona solution, Coffee Arabica), espresso coffee (Cafe La Llave espresso-100% pure coffee, purchased), ground coffee (Nescafe Gold Blend, dark roast, purchased), whole bean coffee (Caffe Verona, purchased). Decaffeinated coffee solution (Pike Place, purchased). No milk/cream or sugar was added to any coffee samples tested in the present study. All coffee products tested were used on the same day of/or day after purchase. Note: We have tested multiple medium roast and dark roast coffee of a variety of types, including, Caffe Verona (dark roast), Pike Place (medium roast), Sirens Blend, Nescafe Gold blend Classico (dark roast) and Sumatra (dark roast) that all show attraction to *C. elegans* . All coffee types used in the present study were purchased from Starbucks coffee shops, and local supermarkets. All coffee used were cooled to around room temperature before use in chemotaxis assays.
